# Do perceived social neighborhood factors explain the association between neighborhood age composition and mental health among Dutch older adults?

**DOI:** 10.1186/s12889-021-11453-w

**Published:** 2021-07-13

**Authors:** Eline Verspoor, Mariëlle A. Beenackers, Joost Oude Groeniger, Frank J. van Lenthe

**Affiliations:** 1 Department of Public Health, Erasmus University Medical Center, Rotterdam, The Netherlands; 2 Department of Geriatric Medicine, Radboud University Medical Center, Nijmegen, the Netherlands; 3 Department of Public Administration and Sociology, Erasmus University, Rotterdam, the Netherlands; 4 Department of Human Geography and Spatial Planning, Utrecht University, Utrecht, the Netherlands

**Keywords:** Neighborhood age structure, Social environment, Age distribution, Mental health

## Abstract

**Background:**

In the light of urbanization and aging, a crucially relevant policy question is how to shape neighborhoods to foster healthy aging. An important debate is whether older adults should group in neighborhoods, or whether a more mixed neighborhood age composition is more beneficial to health and well-being. We therefore assessed the association between neighborhood age structure and mental health and the mediating role of individual perceptions of neighborhood social factors.

**Methods:**

We conducted multivariable linear regression models and causal mediation analyses in 1255 older adults of the Dutch Globe study. The neighborhood age structure was measured in 2011 as the homogeneity of the age composition (using the Herfindahl-Hirschman index, range from 0 to 100, a higher score indicating more homogeneity) and the percentage of specific age groups in a neighborhood. Mental health was measured in 2014 by the Mental Health Inventory-5 score (range 0 to 100, a higher score indicating better mental health). Potential mediators were assessed in 2011 and included perceptions of neighborhood social cohesion, feeling at home in a neighborhood, and social participation.

**Results:**

A more homogeneous age composition (not specified for age) and a higher percentage of children living in a neighborhood were associated with better mental health, the other age categories were not. Social cohesion, feeling at home and social participation did not mediate the associations.

**Conclusions:**

The neighborhood age composition may be an interesting but currently insufficiently understood entry point for policies to improve older adult’s mental health status.

**Supplementary Information:**

The online version contains supplementary material available at 10.1186/s12889-021-11453-w.

## Background

An increasing number of older adults can be expected to live longer independently at home and become more reliant on their social environment. Accordingly, the neighborhood might be increasingly important for older adults’ health and well-being. A crucially relevant policy question is how to shape neighborhoods to foster healthy aging and a relevant debate [1] is whether older adults should group in neighborhoods, or whether a more mixed neighborhood age composition is more beneficial [2]. The neighborhood age composition may affect how people feel about their neighborhood [3], and influence community life, including opportunities for social engagement, social cohesion, loneliness, and social participation. In turn, these individual perceptions of neighborhood social factors may be associated with older adult’s mental health status [3].

Previous studies on the association between the neighborhood age structure and health only assessed the percentage of older adults in a neighborhood and provided mixed results [4, 5]. Some studies found a high percentage of older adults in a neighborhood to be associated with better health outcomes [6, 7], potentially because older adults living in neighborhoods with a high proportion of older adults have more active social ties and social engagement [8]. Others did not observe an association [9, 10], or observed an association with worse health outcomes [4, 11], which may be explained by lower social support from neighbors. A previous study of Cagney et al. (2006) suggests that not only the proportion of older adults but also the neighborhood age composition may be relevant for health [12]. It is thought that a mixed neighborhood age composition, where younger and older age groups live together, may promote healthy aging through individual perceptions of neighborhood social factors such as providing social participation [13–15], and a higher perceived social cohesion [16–18].

So far, the association between the neighborhood age composition and mental health remains unclear. Most previous studies on the neighborhood age composition and health used only cross-sectional data, which limits the interpretation of the results. Moreover, little attention has been paid to underlying pathways in the association between the neighborhood age composition and health. In this study, we aim to assess the association between the neighborhood age composition and mental health of older adults in the Netherlands and to explore important mediating pathways through social (neighborhood) factors.

## Methods

### Study population

A longitudinal sample of respondents participating in the 2011 and 2014 waves of the Dutch population-based cohort study GLOBE (a Dutch acronym for ‘Health and Living Conditions of the Population of Eindhoven and surroundings’) was used. The sampling and design of the GLOBE study are described elsewhere [19]. Briefly, the GLOBE study was initiated in 1991 and invited residents from Eindhoven (The Netherlands) and surrounding cities, aged between 15 and 75 years old. Participants were asked to fill out a survey, and were asked for follow-up in 1997, 2004, 2011 and 2014. In addition to this study sample, a new sample was included for the representativeness of the study sample in 2004 and 2014. In our study, we included all individuals who were included in 1991 or in 2004 with data available in 2011 and 2014. In 2011, respondents residing in Eindhoven and surrounding cities, aged 25 years and over were invited to fill out a survey. In 2011, a total of 3862 persons responded to the survey (response 67.1%). In 2014 all participants were invited again for a follow-up from which 2724 participants responded. The current study is restricted to 1380 respondents that were aged 65 years and over in 2011. For the current study, we excluded respondents with less than three items available on a total of five items to calculate the mental health status in 2014 (*N* = 75) or no valid locational information necessary to link the neighborhood information in 2011 (*N* = 49). Under the Dutch law for medical-scientific research (WMO), ethical approval of this type of non-invasive survey research is not required. The participants were not asked to actively sign an informed consent form but the background and objectives of the study were communicated on the first page of the questionnaire and in the accompanying invitation letter. The completion of the questionnaire was voluntary. The use of personal data in the GLOBE study complies with the Dutch Personal Data Protection Act and the Municipal Database Act and has been registered with the Dutch Data Protection Authority (number 1248943).

### Mental health

Mental health was assessed through the GLOBE survey in 2014, by the 5-item mental health inventory (MHI-5), a validated measure used for the identification of persons with depressive symptoms [20]. The MHI-5 consists of the following five questions: over the last 4 weeks, how often: (I) ‘Have you felt so down in the dumps that nothing could cheer you up?’, (II) ‘Have you felt downhearted and blue?’, (III) ‘Have you been a happy person?’, (IV) ‘Have you been a very nervous person?’ and (V) ‘Have you felt calm and peaceful?’. Each item has six possible responses ranging from ‘all the time’ (1 point) to ‘none of the time’ (6 points). The scores on the answers of the third and fifth questions have been reversed to ensure that a higher item value indicates better mental health. A total mental health score was calculated when at least three out of five questions were answered, by taking the mean of the five items and transforming it to a 0 to 100 points scale to improve interpretation (a higher score indicating better mental health) [21].

### Neighborhood age composition

Data on the distribution of age categories in neighborhoods in the Netherlands were obtained from Statistics Netherlands, which was available on the 1st of January in 2011 [22]. In the Netherlands municipalities are divided into neighborhoods (in Dutch: ‘buurten’) and districts (in Dutch: ‘wijken’). Neighborhoods form the lowest aggregation level and are defined from a building point of view or socio-economic structure whereas districts are a sum of consecutive neighborhoods. Assessing social pathways, such as social cohesion, feeling at home and social participation in a neighborhood, we considered the lowest neighborhood level as the most relevant aggregation level to answer our research question. This aggregation level seems to fit best with the relevant activity space around the homes of older adults. We linked the data on age categories with individual level data from the GLOBE study on ‘neighborhood-codes’ (in Dutch: ‘buurtcodes’). On average, a neighborhood includes approximately 1800–2000 inhabitants. The number of inhabitants of neighborhoods in our study population varied between 80 to 9500 in 2011 [22].

For each neighborhood, the percentage of people in the age categories 0 to 15 years old, 15 to 25 years old, 25 to 45 years old, 45 to 65 years old, and 65 years and older were available. To describe the neighborhood age composition for each respondent, we constructed the Herfindahl-Hirschman Index (HHI); a concentration index indicating the homogeneity of the age composition in a neighborhood. The HHI is a measure of market concentration in economics [23], but can also be used to determine other concentrations such as age concentration. To calculate the HHI, we used the formula below (1) where *Si* is the proportion of the specific neighborhood age group *i* in the total population from the neighborhood and *N* the number of age categories (*N* = 5). To improve interpretation, we multiplied the total by 100 [24].

1 $$ HHI=1-{\sum}_{i-1}^N{Si}^2\ast 100 $$

The theoretical range from the index runs from 0 to 100, with 0 (minimal homogeneity) representing a neighborhood where everyone has a different age category and 100 (maximal homogeneity) representing a neighborhood where everyone is in the same age category [25]. A disadvantage of the HHI is that no distinction can be made in the composition of age groups (e.g. assuming a high HHI, no distinction can be made between a high percentage of older adults or a high percentage of for example young adults). We therefore also assessed the percentages of specific age groups in a neighborhood to unravel the association between the neighborhood age composition and mental health.

### Neighborhood- and individual-level confounders

All individual-level confounders were assessed through the GLOBE survey in 2011. Sex (male and female) and age (in years) were included. Marital status was defined as: married (or partnership), never married; divorced; widowed. Education, defined as the highest attained educational level of the respondent, was classified according to the International Standard Classification of Education (ISCED): high (ISCED 5–7); middle (ISCED 3–4); and low (ISCED 0–2). Household income (not equivalized) was classified as: high (2600 euro per month and higher), moderate (1800–2600 euro per month), intermediate (1200–1800 euro per month), or low (0–1200 euro per month). Neighborhood socioeconomic status was assessed in 2011 by the neighborhood income level, defined as the average disposable personal income per year (*1000 Euro) [22].

### Potential mediators

All potential mediators were assessed through the GLOBE survey in 2011. Four social (neighborhood) perception items were considered, including 1) most people in this neighborhood get on with each other pleasantly, 2) most people in this neighborhood are willing to help each other, 3) I move out of this neighborhood if I get the chance, and 4) I often feel alone in this neighborhood [26, 27]. The items were measured on a 5-point Likert scale response option: from strongly disagree to strongly agree. A principal component analysis with Varimax rotation distinguished two factors (Supplementary File 1, Table S1). The first factor was labeled as (perceived) social cohesion and the second factor was labeled as perceived ‘feeling at home ‘in a neighborhood. For both factors, a standardized factor score (mean of 0 and a standard deviation of 1) was constructed using factor loadings. Additionally, social participation was assessed by a question of whether the person was involved in any cultural or social organization and was dichotomized in ‘yes, being involved in any organization’ and ‘no, not being involved in any organization’.

### Analytical approach

**Fig. 1:**
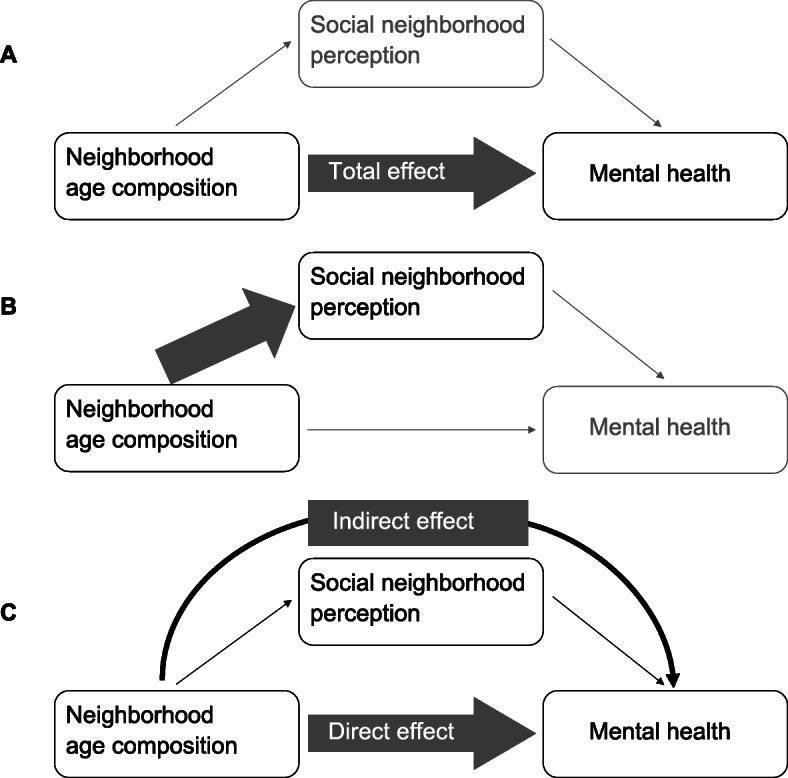
Conceptual model of the association between neighborhood age composition and mental health of older adults. **A** The total effect of the neighborhood age composition on mental health. **B** The association between the neighborhood age composition and social (neighborhood) factors. **C** The direct and indirect effect of the neighborhood age composition on mental health

Characteristics of the study population were described by means and standard deviations (SD) and frequencies and percentages. Multivariable linear regression models were used to assess the relationship between neighborhood age composition and mental health (Fig. 1A). As neighborhood-level variance in mental health status was low, ordinary least squares regression models were used. The association between the exposure and mediators was assessed by linear regression models for (perceived) social cohesion and perceived feeling at home or logistic regression models for social participation (Fig. 1B). In addition, we also assessed the association between mediators and the outcome by linear regression models. We performed causal mediation analysis [28, 29] to examine to what extent the association between the neighborhood age composition and mental health was mediated by individual perceptions of neighborhood social factors (Fig. 1C). We conducted the analyses for each individual perceptions of neighborhood social factors separately (perceived social cohesion, perceived feeling at home, or social participation). One of the main advantages of this mediation method is that it is able to decompose a total effect into direct and indirect effects, even in models with exposure-mediator interactions [29]. The counterfactual definitions of the natural direct and indirect effects factor in this interaction effect represent a population summary of the effects at different levels of the mediator [30]. As the exposure and mediator might interact in their effect on the outcome, we included interaction terms (exposure*mediator) in our models.

All models were adjusted for sex, age, marital status, education, household income, and neighborhood income. We adjusted the models for household income to cover any influence of income on the choice of neighborhood. Therefore household income was not equivalized. In addition, we adjusted our models for neighborhood income to address the level of deprivation of a neighborhood (socio-economic status of a neighborhood). However, since neighborhood income may also be a mediator, instead of a confounder, in the association between the neighborhood age composition and mental health, we also conducted a sensitivity analysis excluding neighborhood income as a confounder [31]. Results were displayed as (unstandardized) beta’s (B) and 95% confidence intervals (95% CI). To impute missing values on the confounders and mediators (2.9% missing values on 7 variables), a multiple imputation procedure was used (*n* = 20 imputation sets). Pooled results from the 20 imputed datasets were used for analyses [32], whereas non-imputed data were used to describe characteristics of the study population. Statistical analyses were executed using Stata Version 13.

## Results

### Characteristics of the study population

Among 1255 respondents, 51% was female and the mean age was 73 (SD 6) years (Table 1). The mean mental health score was 73 (SD 15) points. Of all respondents, 70% was married or in a relationship, and 19% was widowed. The majority of the population (53%) was low educated. A total of 74% of the older adults participated in a cultural or social organization. The mean neighborhood age composition score was 77 on a scale from 0 to 100 where a higher score indicates more homogeneity. The mean percentage of the number of children, adolescents, young adults, middle-aged adults, and older adults in all neighborhoods was respectively: 16, 12, 26, 27, and 20%. The average neighborhood income was 30,000 (SD 7000) Euro per year.

**Table 1:** Characteristics of the study population

Characteristics	Total (*N* = 1255)
**Individual-level**	
MHI score, mean (SD)^a^	73 (15)
Sex, female	51.3%
Missing	1.4%
Age in years, mean (SD)	73 (6)
Missing	0.0%
Marital status	
Married or partnership	70.0%
Never been married	3.6%
Divorced	6.4%
Widowed	19.4%
Missing	0.7%
Education	
Low	53.4%
Middle	15.7%
High	24.1%
Missing	6.8%
Household income (monthly)	
≥ €2600	25.7%
€1800–2600	29.6%
€1200–1800	21.8%
< €1200	9.3%
Missing	13.5%
Social participation	
Yes	74.1%
No	25.9%
Missing	0.0%
**Neighborhood-level**	
Number of neighborhoods	215
Number of respondents in the neighborhood	1–53
HHI score, mean (SD)^b^	77 (2)
Percentage of children (0–14 years), mean (SD)	16 (4)
Percentage of adolescents (15–24 year), mean (SD)	12 (3)
Percentage of young adults (25–44 year), mean (SD)	26 (7)
Percentage of middle-aged adults (45–65 year), mean (SD)	27 (5)
Percentage of older adults in the neighborhood (65+ year), mean (SD)	20 (9)
Missing	0.0%
Neighborhood income level, mean (SD) * 1000 Euro (yearly)	30 (7)
Missing	0.2%

*MHI* mental health 5-inventory, *SD* standard deviation, *HHI* Herfindahl-Hirschman Index. ^a^ The MHI is assessed by the 5-item mental health inventory (MHI-5), range from 0 to 100 (a higher score indicating better mental health) ^b^ The HHI is defined as homogeneity of the neighborhood age structure (score from 0 to 100, where a higher score indicates more homogeneity in neighborhood age structure). The percentages of specific age groups in a neighborhood can be interpreted as the mean percentage of all 215 neighborhoods. The neighborhood income level was defined as the average disposable personal income (*1000 Euro). The neighborhood income level can be interpreted as the mean neighborhood income level from all 215 neighborhoods. Individual perceptions of social neighborhood factors (‘social cohesion’ and ‘feeling at home’) were not included in this table because they were standardized factor scores (mean of 0, standard deviation of 1). The mean and standard deviations for the individual items that were used to construct the factor scores can be found in Supplementary File 1, Table S1

### Neighborhood age composition and mental health

A neighborhood with a more homogeneous age composition (dominant age category is unspecified) was associated with better mental health (B 0.40, 95% CI 0.05; 0.74) (Table 2). Also, participants residing in a neighborhood with a higher percentage of 0–14-year-old children reported a better mental health status (B 0.30, 95% CI 0.08; 0.51). Residing with a higher percentage of adolescents, young adults, middle-aged adults, or older adults in a neighborhood was not significantly associated with mental health. The homogeneity of the neighborhood age composition and the percentage children living in a neighborhood were not associated with individual perceptions of neighborhood social factors (Table 3). Residents living in neighborhoods with a higher percentage of adolescents (OR 0.95, 95% CI 0.90; 0.99) and young adults (OR 0.98, 95% CI 0.96; 1.00) reported somewhat less social participation. Also, residents living in neighborhoods with a higher percentage of young adults felt slightly less at home in their neighborhood (B -0.01, 95% CI -0.02; − 0.00), whereas residents living in neighborhoods with a higher percentage of older adults felt slightly more at home in their neighborhood (B 0.01, 95% CI 0.00;0.01). Furthermore, a higher percentage of middle-aged residents in a neighborhood was associated with slightly more perceived social cohesion (B 0.02, 95% CI 0.00; 0.03).

**Table 2:** Linear regression analysis of the neighborhood age composition with mental health in older adults (*N* = 1255)

	Mental health^**a**^	
Characteristics	B	95% CI
HHI score^b^, homogeneity neighborhood age structure	**0.40**	0.05; 0.74*
Percentage children (0–14 years), mean (SD)	**0.30**	0.08; 0.51*
Percentage adolescents (15–24 year), mean (SD)	0.16	− 0.13; 0.45
Percentage young adults (25–44 year), mean (SD)	− 0.07	− 0.21; 0.06
Percentage middle-aged adults (45–65 year), mean (SD)	0.16	− 0.02; 0.35
Percentage older adults in neighborhood (65+ year), mean (SD)	−0.09	− 0.18; 0.01

*CI* confidence interval, *HHI* Herfindahl-Hirschman Index. ^a^ The MHI is assessed by the 5-item mental health inventory (MHI-5), range from 0 to 100 (a higher score indicating better mental health) ^b^ The HHI is defined as homogeneity of the neighborhood age structure (score from 0 to 100, where a higher score indicates more homogeneity in neighborhood age structure). All models were adjusted for sex, age, marital status, highest attained education, household income, and neighborhood income. * significant at a level of < 0.05

**Table 3:** Association between neighborhood age composition and individual perceptions of neighborhood social factors in older adults (*N* = 1255)

	Social cohesion^**a**^		Feeling at home^**b**^		Social participation^**c**^	
Characteristics	B	95% CI	B	95% CI	OR	95% CI
HHI score^d^, homogeneity neighborhood age structure	−0.02	− 0.04; 0.01	0.01	− 0.01; 0.04	0.97	0.92; 1.03
Percentage children (0–14 years), mean (SD)	−0.00	− 0.02; 0.01	−0.01	− 0.03; 0.01	0.99	0.95; 1.02
Percentage adolescents (15–24 year), mean (SD)	−0.02	− 0.04; 0.01	−0.01	− 0.03; 0.02	**0.95**	0.90; 0.99*
Percentage young adults (25–44 year), mean (SD)	−0.00	− 0.01; 0.01	**−0.01**	− 0.02; − 0.00*	**0.98**	0.96; 1.00*
Percentage middle-aged adults (45–65 year), mean (SD)	**0.02**	0.00; 0.03*	0.00	−0.01; 0.02	1.00	0.97; 1.04
Percentage older adults in neighborhood (65+ year), mean (SD)	0.00	−0.01; 0.01	**0.01**	0.00; 0.01*	1.02	1.00; 1.04

*CI* confidence interval, *HHI* Herfindahl-Hirschman Index. ^a,b^ Social cohesion and feeling at home represented the factor scores and linear regession analyses were carried out. ^c^ Reference for social participation is not participating in any organization. A logistic regression analysis was carried out. ^d^ The HHI is defined as homogeneity of the neighborhood age structure (score from 0 to 100, where a higher score indicates more homogeneity in neighborhood age structure). All models were adjusted for sex, age, marital status, highest attained education, household income, and neighborhood income. * significant at a level of < 0.05

### Mediators and mental health

A higher social cohesion in the neighborhood and feeling at home in a neighborhood were significantly associated with better mental health among older adults. More social participation was not associated with the mental health status of older adults (Supplementary File 1, Table S2).

Based on Tables 2 and 3, no mediation was expected. The mediation analysis showed no mediation, except for the association between the percentage of young adults and mental health of older adults, this association was mediated by the feeling at home in a neighborhood. Results from the mediation analysis can be found in Supplementary File 1, Table S3.

Sensitivity analyses excluding neighborhood income as confounder from the analyses slightly altered the point estimates and confidence intervals but did not change the direction or significance of the associations (Supplementary File 1, Table S4).

## Discussion

In this study, a more homogeneous neighborhood age composition and a higher percentage children aged between 0 and 14 years old in a neighborhood were associated with better mental health 3 years later. Living in a neighborhood with a high percentage of adolescents, young adults, middle-aged adults, or older adults was not associated with the mental health of older adults. Although some small associations were observed, between the neighborhood age composition factors and individual perceptions of neighborhood social factors, these associations were so small that they were deemed irrelevant. Furthermore, our study showed that a better perceived social cohesion and the perceived feeling at home in the neighborhood were associated with a better mental health status among older adults. A higher social participation, involved in any cultural or social organizations, was not associated with the mental health status of older adults. Moreover, we found some mediation effects although no direct effect was observed. Therefore, these results should be interpreted with caution.

Our study is the first study that assessed the influence of other age groups, such as the percentage of young children in a neighborhood, on older adults’ mental health status. Although the results have to be interpreted with caution, our finding that more children in the neighborhood can benefit older adults’ mental health may justify further research into the topic. Better integration of generations is seen as a way to counter ageism in society [1]. Intergenerational opportunities may enrich the experience for all ages; older people pass on traditional practices and knowledge and experiences, while younger people offer information about newer practices and help older people navigate in a rapidly changing society [1]. Although we assessed social (neighborhood) pathways, we did not observe that these pathways mediated this association. To unravel possible pathways, a qualitative study is suggested where older adults in different neighborhoods can be interviewed to assess the importance of the neighborhood age structure on their mental health status.

Our study adds to the knowledge on the association between the neighborhood age composition and mental health, as to the best of our knowledge only one cross-sectional study is known on the neighborhood age structure and mental health. The previous study showed an association of a higher percentage of older adults in a neighborhood with better mental health among older adults [6]. Our study did not confirm this research finding which may be explained by our relatively high mean (20%) and a wide range from 4 to 79% of older adults living in a neighborhood. Whereas the study of Kubzansky [6] had a mean of 13% and a small range of older adults living in a neighborhood from 2 to 23%. It might be that the percentage of older adults does not matter when all neighborhoods have at least a certain percentage of older adults. This might explain why we did not find an association in our study.

Using causal mediation analyses, we did not find evidence for a mediating role of social (neighborhood) factors, including perceived social cohesion, perceived feeling at home, and social participation. Other possible pathways might explain the observed association between the neighborhood age composition and mental health, including participation in social activities that were not in our study (clubs/associations, babysitting, sports, events, etc.), social support, vandalism, or graffiti in a neighborhood. We suggest further research into more diverse neighborhoods in terms of age composition to determine whether and what the most optimal neighborhood age composition is in a neighborhood for healthy aging, accompanied by research into a broader range of potential mediating factors to clarify the possible pathways in the association between the neighborhood age composition and mental health.

### Study limitations and -strengths

To date, studies on the neighborhood age structure focused only on the percentage of older adults in a neighborhood, rather than considering the overall age composition of a neighborhood. Our study builds on previous work, by using both the Herfindahl-Hirschman Index to measure age composition and percentages of different age groups, including children. Also, we explored potential underlying pathways in the association between the neighborhood age composition and mental health by assessing the role of individual perceptions of neighborhood social factors by causal mediation analyses. Nevertheless, our results should be interpreted in light of several limitations. First, there could be selection bias in our study as persons with a low mental health status might have been less likely to participate. This resulted in an overestimation of the mental health status of our study participants, and in turn a potential underestimation of the studied associations. Second, our study assessed neighborhood characteristics at the lowest aggregation level as we considered this as most relevant to answer our research question related to social factors at neighborhood level. This aggregation level seems to fit best with the relevant activity space of older adults. Other aggregation levels (e.g. looking at larger districts or areas or even at the city level) might be relevant as well and may provide alternate conclusions. However, a comparison on aggregation levels was beyond the scope of our study. Third, although we expected neighborhood variation in our mental health outcome due to neighborhood deprivation, neighborhood variation was limited in our sample which may indicate a lack of neighborhood influence on the mental health status of older adults. However, since some neighborhood factors may be positively and others may be negatively associated with mental health of older adults, neighborhood influences cannot be ruled out. Our results require follow-up research, especially since the age composition of a neighborhood is considered in the World Health Organization age-friendly cities policy document [1]. Fourth, although the neighborhood age composition and the mediators were measured 3 years before mental health, we cannot rule out reverse causality where previous mental health status has affected the choice of neighbourhood or, more likely, the perceived social (neighborhood) factors. However, we chose not to adjust for mental health status in 2011 because that may result in an overadjustment. As many older adults have already lived in their neighborhood for a long time, and if we expect that neighborhood factors affect their mental health status, adjusting for mental health in 2011 would overcontrol for the potential influence of the neighborhood factors on mental health. This would in turn underestimate the association between the neighborhood age structure and mental health of older adults. We recommend additional longitudinal research to assess causal associations, looking into changes both in the neighborhood age composition and mental health. Moreover, in this epidemiological study we did not directly assess whether different age groups interacted with each other, to obtain more insight in possible pathways qualitative research can unravel mechanisms more in depth. Fifth, the neighborhood characteristics were measured on the individual level, they were perceptions of individuals on factors related to their neighborhood. For further research we suggest to assess whether factors at the neighborhood-level show an association with the mental health status of older adults. Sixth, although the HHI goes beyond simple percentages, it is still limited since it cannot distinguish between a high percentage of older adults or a high percentage of other age groups in the neighborhood. However, by assessing both the HHI and percentages of different age groups, our study contributes to a better understanding of how the neighborhood age composition is related to mental health.

## Conclusions

Our study is the first study that assessed the association of the neighborhood age structure as the presence of different neighborhood age groups (including children, adolescents, young adults, middle-aged adults, and older adults) on older adults’ mental health status. Moreover, the current study adds to the wider body of literature by assessing possible pathways of individual perceptions of social neighborhood factors in the association between the neighborhood age structure and mental health status among older adults. A more homogeneous neighborhood age structure and more children living in a neighborhood were associated with better mental health status. Although individual perceptions of neighborhood social factors did not explain these associations, our study indicates that the neighborhood age composition is a potentially interesting but still insufficiently understood entry point for policies addressing the challenge of growing urban and aging European cities. Further longitudinal research on the neighborhood age composition and mental health are needed, including research on other possible underlying mechanisms.

## Supplementary Information

**Additional file 1: Table S1.** Rotated factor loadings of individual perceptions of neighborhood social factors (*N* = 1092). **Table S2.** Linear regression analysis of the individual perceptions of social neighborhood factors with mental health in older adults (*N* = 1255). **Table S3.** Mediation analysis between the neighborhood age composition and mental health in older adults of individual perceptions of neighborhood social factors (*N* = 1255). **Table S4.** Sensitivity analysis: Mediation analysis between the neighborhood age composition and mental health in older adults of individual perceptions of neighborhood social factors without neighborhood income as a confounder (*N* = 1255).

## Data Availability

The datasets used and/or analyzed during the current study are available from the corresponding author on reasonable request.

## Abbreviations

GLOBE: Dutch acronym for ‘Health and Living Conditions of the Population of Eindhoven and surroundings’
MHI-5: Five-item mental health inventory
HHI: Herfindahl-Hirschman Index
ISCED: International Standard Classification of Education
SD: Standard deviations
95% CI: 95% confidence intervals
