# Supplementary material for: Do perceived social neighborhood factors explain the association between neighborhood age composition and mental health among Dutch older adults?
Source: BMC Public Health. 2021 Jul 13;21:1390. doi: 10.1186/s12889-021-11453-w (PMC8278722; doi:10.1186/s12889-021-11453-w)
Supplement: Supplementary file 1 — Additional file 1: Table S1. Rotated factor loadings of individual perceptions of neighborhood social factors (N = 1092). Table S2. Linear regression analysis of the individual perceptions of social neighborhood factors with mental health in older adults (N = 1255). Table S3. Mediation analysis between the neighborhood age composition and mental health in older adults of individual perceptions of neighborhood social factors (N = 1255). Table S4. Sensitivity analysis: Mediation analysis between the neighborhood age composition and mental health in older adults of individual perceptions of neighborhood social factors without neighborhood income as a confounder (N = 1255). [file 12889_2021_11453_MOESM1_ESM.docx]

**Do perceived social neighborhood factors explain the association between neighborhood age composition and mental health among Dutch older adults?**

**Supplementary File 1**

Eline Verspoor ^a,b^

Mariëlle A. Beenackers ^a^

Joost Oude Groeniger ^a,c^

Frank J. van Lenthe ^a,d^

^a^ Department of Public Health, Erasmus University Medical Center, Rotterdam, the Netherlands

^b^ Department of Geriatric Medicine, Radboud University Medical Center, Nijmegen, the Netherlands

^c^ Department of Public Administration and Sociology, Erasmus University, Rotterdam, the Netherlands

^d^ Department of Human Geography and Spatial Planning, Utrecht University, the Netherlands

**Corresponding author:** Dr. M.A. Beenackers, Department of Public Health, Erasmus University Medical Center, PO Box 2040 CA Rotterdam, the Netherlands. E-mail address: [M.Beenackers@erasmusmc.nl](mailto:M.Beenackers@erasmusmc.nl)

**Table S1.** Rotated factor loadings of individual perceptions of neighborhood social factors (N=1092)

| **Items ^a^** |  | **Factor loadings ^b^** |  |
| --- | --- | --- | --- |
|  | **Mean (SD)** | **Social cohesion** | **Feeling at home** |
| Most people in this neighborhood get on with each other pleasantly | 3.97 (0.92) | **0.95** | 0.13 |
| Most people in this neighborhood are willing to help each other | 3.92 (0.91) | **0.96** | 0.07 |
| I move out of this neighborhood if I get the chance ^c^ | 4.45 (0.91) | 0.08 | **0.82** |
| I often feel alone in this neighborhood ^c^ | 4.06 (0.91) | 0.10 | **0.81** |
| **Explained variance** |  | **45.6%** | **79.3%** |

^a^ Answering categories ranged from (1) ‘totally disagree’ to (5) ‘totally agree’

^b^ Bold factor loadings are the most important items within the factors

^c^ Recoded so answering categories ranged from (1) ‘totally agree’ to (5) ‘totally disagree’

**Table S2.** Linear regression analysis of the individual perceptions of social neighborhood factors with mental health in older adults (N=1255)

|  | **Mental health^a^** | |
| --- | --- | --- |
| **Characteristics** | **B** | **95% CI** |
| **Social cohesion** | **1.22** | **0.37; 2.07*** |
| **Feeling at home** | **3.76** | **2.92; 4.61*** |
| **Social participation** | -0.13 | -2.00; 1.74 |

CI: confidence interval. The MHI is assessed by the 5-item mental health inventory (MHI-5), range from 0 to 100 (a higher score indicating better mental health)
* significant at a level of <0.05. All models were adjusted for Herfindahl-Hirschman Index sex, age, marital status, highest attained education, household income, and neighborhood income.

**Table S3.** Mediation analysis between the neighborhood age composition and mental health in older adults of individual perceptions of neighborhood social factors (N=1255)

|  | **Proportion mediated, %** | **P-value for the indirect effect** |
| --- | --- | --- |
| **HHI score**^a^**, homogeneity neighborhood age structure** |  |  |
| Social cohesion | 4 | 0.26 |
| Feeling at home | 12 | 0.24 |
| Social participation | 1 | 0.73 |
| **Percentage of children (0 to 14 year) in a neighborhood** |  |  |
| Social cohesion | 2 | 0.52 |
| Feeling at home | ^b^ | - |
| Social participation | ^b^ | - |
| **Percentage of adolescents (15 to 24 year) in a neighborhood** |  |  |
| Social cohesion | ^b^ | - |
| Feeling at home | ^b^ | - |
| Social participation | ^b^ | - |
| **Percentage of young adults (25 to 44 year) in a neighborhood** |  |  |
| Social cohesion | 7 | 0.44 |
| Feeling at home | **60** | **0.01** |
| Social participation | ^b^ | - |
| **Percentage middle-aged older adults (45 to 65 year) in a neighborhood** |  |  |
| Social cohesion | 6 | 0.42 |
| Feeling at home | 6 | 0.64 |
| Social participation | 0 | 0.87 |
| **Percentage of older adults (65+ year) in a neighborhood** |  |  |
| Social cohesion | ^b^ | - |
| Feeling at home | ^b^ | - |
| Social participation | 3 | 0.82 |

CI: confidence interval. HHI: Herfindahl-Hirschman Ondex. ^a^ The HHI is defined as homogeneity of the neighborhood age structure (score from 0 to 100, where a higher score indicates more homogeneity in neighborhood age structure). ^b^ Negative proportion as a result of an indirect effects that is in the opposite direction of the total effect.

All models were adjusted for sex, age, marital status, highest attained education, household income, and neighborhood income.

**Table S4.** Sensitivity analysis: Mediation analysis between the neighborhood age composition and mental health in older adults of individual perceptions of neighborhood social factors without neighborhood income as a confounder (N=1255)

|  | **Proportion mediated, %** | **P-value for the indirect effect** |
| --- | --- | --- |
| **HHI score^a^, homogeneity neighborhood age structure** |  |  |
| Social cohesion | ^b^ | - |
| Feeling at home | 9 | 0.40 |
| Social participation | ^b^ | - |
| **Percentage of children (0 to 14 year) in a neighborhood** |  |  |
| Social cohesion | ^b^ | - |
| Feeling at home | ^b^ | - |
| Social participation | ^b^ | - |
| **Percentage of adolescents (15 to 24 year) in a neighborhood** |  |  |
| Social cohesion | ^b^ | - |
| Feeling at home | ^b^ | - |
| Social participation | ^b^ | - |
| **Percentage of young adults (25 to 44 year) in a neighborhood** |  |  |
| Social cohesion | 14 | 0.18 |
| Feeling at home | **69** | **<0.01** |
| Social participation | ^b^ | - |
| **Percentage middle-aged older adults (45 to 65 year) in a neighborhood** |  |  |
| Social cohesion | 9 | 0.39 |
| Feeling at home | 18 | 0.23 |
| Social participation | 1 | 0.69 |
| **Percentage of older adults (65+ year) in a neighborhood** |  |  |
| Social cohesion | ^b^ | - |
| Feeling at home | ^b^ | - |
| Social participation | 3 | 0.82 |

CI: confidence interval. HHI: Herfindahl-Hirschman Index. a The HHI is defined as homogeneity of the neighborhood age structure (score from 0 to 100, where a higher score indicates more homogeneity in neighborhood age structure). b Negative proportion as a result of an indirect effects that is in the opposite direction of the total effect. All models were adjusted for sex, age, marital status, highest attained education, and household income..
